# From data to action: a community-centered framework for translating community health assessment into coordinated public health strategy

**DOI:** 10.3389/fpubh.2026.1879850

**Published:** 2026-07-10

**Authors:** Diego R. Hijano

**Affiliations:** 1 Department of Infectious Diseases, St. Jude Children's Research Hospital, Memphis, TN, United States; 2 Department of Pediatrics, University of Tennessee Health Science Center, Memphis, TN, United States

**Keywords:** community health assessment, cross-sector collaboration, health equity, integrated care, population health, public health infrastructure, social determinants of health

## Abstract

Public health agencies routinely conduct community health assessments, yet many jurisdictions continue to face challenges translating population health data into coordinated action that addresses structural determinants of health, health equity, and public health infrastructure needs. These challenges became increasingly visible during the COVID-19 pandemic and highlight the need for community-centered approaches that align public health, healthcare systems, and community partnerships across sectors. This Policy and Practice Review presents a community-centered framework for translating local population health data into coordinated, equity-oriented population health strategy. Using Shelby County, Tennessee, a large urban county in the southern United States, as an illustrative case, the analysis synthesizes publicly available datasets, community health needs assessments, and institutional reports to align local priorities, governance structures, and cross-sector operational strategies within a unified implementation approach. The framework organizes population health priorities into five domains: chronic disease prevention, maternal and child health equity, HIV and sexual health, behavioral health and substance use, and violence prevention and community safety. Across domains, the framework emphasizes shared measurement, data integration, community partnerships, trust, and cross-sector accountability as foundational components of sustainable population health improvement. Although not an implementation evaluation, this review offers a practical approach for jurisdictions seeking to strengthen public health infrastructure, operationalize equity-centered decision-making, and move from fragmented programs toward coordinated population health action responsive to evolving community needs.

## 1 Introduction

Public health agencies in many settings face a persistent paradox: they possess substantial clinical and community resources; however, population-level outcomes remain uneven, with stark inequities across neighborhoods and demographic groups (1, 2). Numerous jurisdictions have documented differences in life expectancy, infant mortality, and chronic disease burden that reflect deep structural and social determinants of health (3, 4). Community health assessments routinely describe these patterns, but translation of assessment findings into coordinated, system-level action remains limited. The COVID-19 pandemic further exposed how fragmented governance, parallel service delivery structures, limited community trust, and inconsistent data-sharing practices can constrain the ability of public health systems to respond effectively to upstream determinants and evolving community needs (5–10).

This persistent gap between assessment and action has generated increasing interest in population health frameworks that link data, policy, community partnerships, and implementation (8, 11, 12). Such frameworks aim to integrate public health, healthcare, and community sectors through shared priorities, cross-sector governance, measurable equity-oriented outcomes, and sustained community engagement (6, 10, 13–17). However, many existing models are insufficiently operationalized or not adapted to the realities of local implementation, limiting their ability to guide coordinated decision-making, build community trust, and translate assessment findings into durable public health practice.

This paper addresses this challenge by proposing a community-centered framework for translating community health data into coordinated population health action, illustrated through the example of Shelby County, Tennessee, a large urban county in the southern United States. The county reflects demographic diversity, entrenched socioeconomic inequities, and persistent disparities across chronic disease, maternal and child health, behavioral health, and injury, conditions common to many urban jurisdictions (18–22). By synthesizing publicly available data sources and institutional reports, this analysis develops a structured approach to organizing community health priorities, aligning system capacity, strengthening cross-sector partnerships, and guiding policy decisions toward sustained, equity-centered population health improvement (8, 13).

## 2 Assessing community health priorities and system fragmentation

### 2.1 Development of the community-centered framework

This Policy and Practice Review used a structured analytic approach to develop a community-centered framework for translating community health assessment findings into coordinated population health action. The framework was informed through synthesis of publicly available epidemiologic datasets, community health needs assessments, institutional reports, and public-facing strategic documents relevant to population health planning and public health practice. Most reports and datasets were published between 2022 and 2025 and were selected based on their relevance to population health outcomes, health equity, public health infrastructure, and community health planning within Shelby County, Tennessee. Framework development proceeded in three stages.

First, publicly accessible datasets and reports were reviewed to identify major contributors to premature mortality, morbidity, and health inequities within Shelby County, Tennessee. Sources were selected based on relevance to population health outcomes and included state and local health department datasets, national benchmarking tools, vital statistics reports, and community health needs assessments conducted by regional healthcare systems (19, 20, 23–25). These materials provided an empirical foundation for identifying population-level patterns, structural determinants of health, and disparities across neighborhoods and demographic groups (1, 4).

Second, publicly available program descriptions, institutional strategic plans, and community initiative reports from health and social service organizations were examined to assess how existing activities aligned with identified population health needs. A directed qualitative document review approach was used to identify recurring themes related to community partnerships, prevention priorities, governance structures, service integration, and implementation gaps across sectors (24–30). The analysis focused on alignment between operational activities and shared population health goals rather than evaluation of program effectiveness. This approach reflects the practical realities faced by many jurisdictions, where strengthening coordination across existing systems may be more feasible than creating entirely new infrastructure (5, 7).

**Figure 1:**
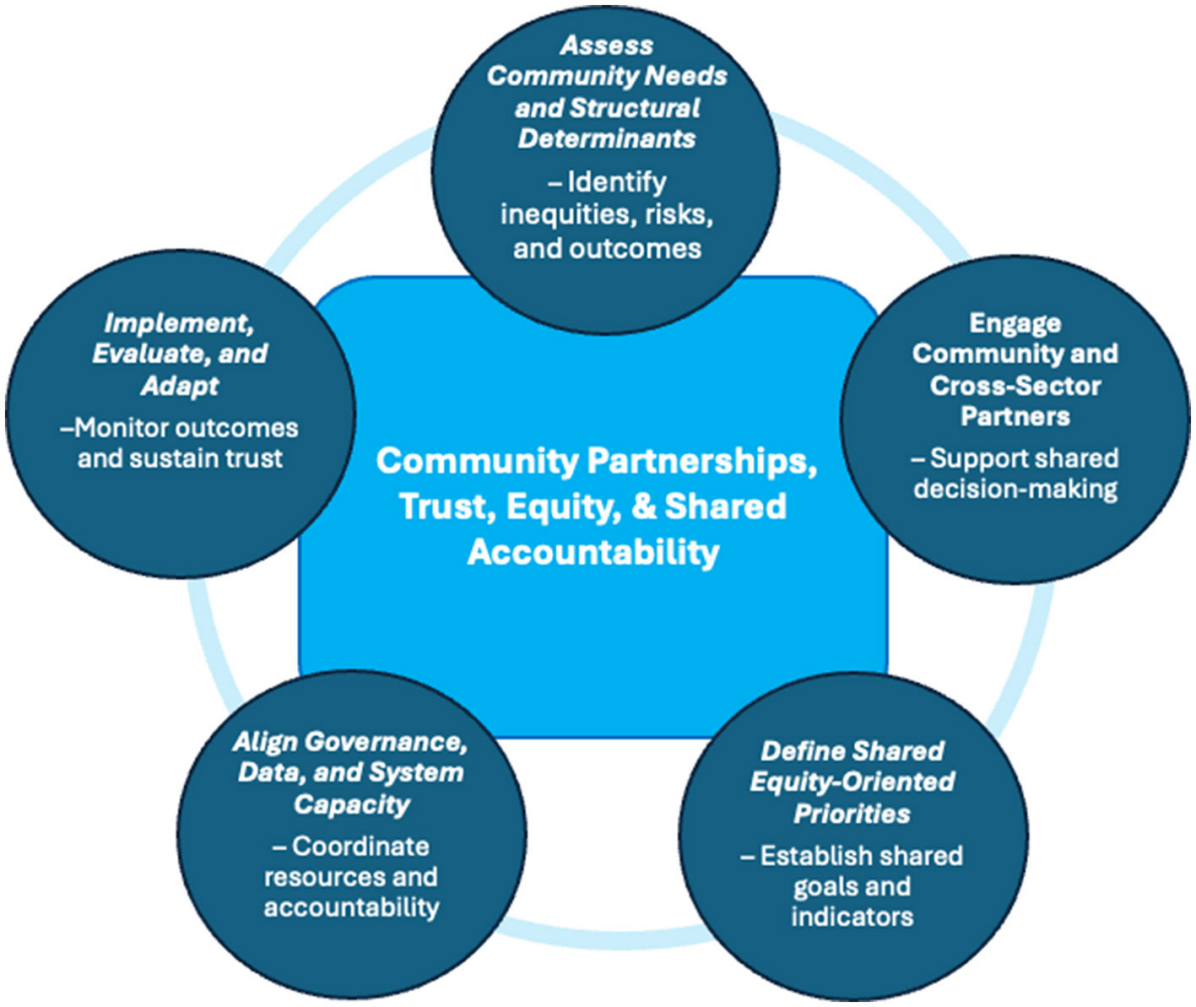
Community-centered framework for translating community health assessment into coordinated public health action. This figure illustrates an iterative framework for translating community health data into a coordinated, equity-oriented population health strategy through sustained collaboration among public health agencies, healthcare systems, community organizations, and affected communities. The model includes five interconnected stages: assessing community health needs and structural determinants; engaging community and cross-sector partners in shared decision-making; defining shared equity-oriented priorities; aligning governance, data systems, and organizational capacity; and implementing, evaluating, and adapting strategies using indicators that can be disaggregated by race, income, and geography. The continuous loop reflects the dynamic nature of population health planning, in which changes in population needs, institutional capacity, community trust, and equity outcomes require ongoing reassessment rather than one-time, linear implementation. Community partnerships, trust, equity, and shared accountability are positioned at the center to emphasize that effective population health strategies depend on sustained collaboration with affected communities and ongoing attention to distributional impacts across populations and neighborhoods.

Third, findings from the epidemiologic and document review phases were synthesized into a five-step framework encompassing: (1) assessment of population health data, (2) cross-sector stakeholder engagement, (3) definition of shared priorities, (4) alignment of governance and system capacity, and (5) implementation, monitoring, and evaluation. Particular attention was given to the role of community partnerships, trust, shared accountability, and equity-oriented measurement in supporting coordinated population health strategy (15, 16, 31–33). Figure 1 illustrates the iterative framework used to organize the proposed approach.

### 2.2 Identification of priority population health domains

To identify priority domains for inclusion in the framework, community health needs assessments and institutional reports were synthesized using directed content analysis, a qualitative approach in which existing theory or prior research guides the development of initial coding categories (34). Initial categories were informed by population health, integrated care, public health governance, and collective impact literature and included population health priorities, governance structures, cross-sector partnerships, implementation strategies, and measurement approaches. Coding and synthesis were conducted by a single reviewer; therefore, formal assessment of inter-coder agreement and adjudication of coding disagreements were not applicable. Themes that did not align with the initial categories were incorporated through iterative refinement of the coding framework. Sources included assessments and reports from regional hospital systems, local public health agencies, non-profit organizations, and community-based initiatives addressing domains such as housing, nutrition, maternal health, behavioral health, HIV prevention, and violence prevention (20, 21, 24–28, 30, 35–39).

Across documents, recurring themes related to population health priorities, implementation barriers, and cross-sector coordination needs were systematically extracted, coded, and grouped into conceptual categories. These categories informed the development of the framework's five population health priority domains.

### 2.3 Shared measurement, data integration, and accountability

To support future operationalization, the framework incorporates a structured approach to shared measurement and cross-sector data integration. Illustrative indicators were identified from existing surveillance systems and routinely collected administrative data sources, including state vital statistics, hospital discharge databases, behavioral risk surveys, trauma registries, and community program metrics (20, 21, 23–26, 30, 35).

Indicator selection prioritized measures that: (1) are routinely collected and feasible for local implementation; (2) can be disaggregated by race, income, and geography to support equity assessment; and (3) capture both health outcomes and upstream social determinants of health (3, 9, 13, 22, 40, 41).

The resulting indicator set informed the framework's measurement and accountability components and is summarized in Table 1.

**Table 1:** Illustrative indicators and representative data sources for monitoring implementation and equity across priority population health domains within a population health framework.

Priority domain	Illustrative indicators	Potential data sources
Chronic disease prevention	Hypertension and diabetes control rates; obesity prevalence; proportion of adults meeting physical activity guidelines; neighborhood-level access to healthy food and safe physical activity spaces; participation in community-based nutrition or lifestyle programs	Electronic health records from primary care networks; Behavioral Risk Factor Surveillance System (BRFSS); local health department surveillance reports; community organization and public health partnership data (13–15, 18, 19)
Maternal and child health equity	Infant mortality and preterm birth rates; maternal hypertension; postpartum follow-up rates; timely access to prenatal care by race and geography; housing stability among pregnant or postpartum individuals	Vital statistics records; Pregnancy Risk Assessment Monitoring System (PRAMS); hospital discharge data; housing, social service, and community support program data (13–15, 18–20, 27)
HIV and sexual health	New HIV diagnoses; pre-exposure prophylaxis uptake; viral suppression among persons living with HIV; HIV testing coverage in community and primary care settings; community-based HIV outreach engagement	State and local HIV surveillance registries; Ryan White program data; community health center quality reports; pharmacy dispensing data for pre-exposure prophylaxis; community-based organization reports (13–15, 18, 21, 24, 26, 34, 35)
Behavioral health and substance use	Overdose mortality; initiation of substance use treatment; emergency or crisis service utilization; integration of behavioral health within primary care; access to crisis response and harm reduction services	Medical examiner and vital records data; state behavioral health or substance use treatment databases; emergency medical services call records; hospital emergency department discharge data; community behavioral health program reports (13–15, 18, 23, 26)
Violence prevention and community safety	Homicide and assault-related injury rates; firearm-related hospitalizations; repeat victimization; participation in community-led violence prevention initiatives	Hospital trauma registry data; law enforcement incident reports; emergency department injury surveillance; community organization and neighborhood program data (13–15, 18, 23, 28, 29)

### 2.4 Priority domains for coordinated population health action

Application of this analytic approach identified five priority domains for coordinated population health action: chronic disease prevention, maternal and child health equity, HIV and sexual health, behavioral health and substance use, and violence prevention and community safety. These domains reflect recurrent contributors to premature mortality, inequity, and community-level health burden identified across local epidemiologic assessments and institutional reports (19–21, 23–25).

Within each domain, population health priorities are aligned with illustrative operational activities across public health agencies, healthcare systems, and community-based organizations, demonstrating how community health assessment findings can be translated into coordinated areas of action (Table 2). Complementing this structure, a set of illustrative indicators derived from routinely collected public health and administrative data provides a practical approach to shared measurement, accountability, and equity-oriented monitoring across sectors (Table 1) (19–21, 23).

**Table 2:** Illustrative alignment of strategic population health priorities with cross-sector operational strategies to support coordinated implementation and accountability.

Population health priority (strategic)	Cross-sector operational strategies
Reduce preventable mortality and chronic disease through community-based prevention and health system integration	Coordinate chronic disease prevention, tobacco cessation, nutrition support, and community-based lifestyle interventions across healthcare and community settings
Advance maternal and child health equity by addressing social and environmental determinants	Integrate prenatal care, maternal risk reduction, home visiting, and social support services through healthcare and community partnerships
Reduce HIV and sexual health disparities through integrated, stigma-free prevention and care	Expand HIV and sexually transmitted infection testing, pre-exposure prophylaxis access, and culturally responsive outreach through clinical and community-based partnerships
Reduce substance use and mental health–related morbidity through trauma-informed care and integration with primary care	Align behavioral health services, overdose prevention, crisis response, and harm reduction initiatives across healthcare and community systems
Prevent violence and improve community safety through upstream and community-based interventions	Coordinate violence prevention, youth development, hospital-based intervention, and neighborhood safety initiatives through cross-sector collaboration
Cross-cutting: strengthen public health infrastructure through data integration, partnerships, and community engagement	Develop shared data systems, evaluation capacity, governance structures, and community partnerships to support accountability and continuous improvement

Figure 1 illustrates the proposed five-step framework through which population health data, community partnerships, cross-sector stakeholder engagement, shared priority-setting, governance alignment, and implementation, monitoring, and evaluation are integrated into an iterative population health strategy. The framework emphasizes that sustained population health improvement depends not only on identifying health priorities, but also on strengthening trust, coordination, and shared accountability among institutions and affected communities (10, 15, 16, 32).

## 3 Policy and practice applications across priority domains

The following sections illustrate how the proposed framework can be applied across major population health priority areas commonly identified in community health assessments and public health planning processes. Although each domain reflects distinct epidemiologic patterns and operational challenges, several cross-cutting themes emerged consistently across sectors, including fragmented governance structures, limited integration between clinical and community-based services, inconsistent data-sharing practices, and insufficient alignment between population-level priorities and operational implementation strategies. Across domains, the framework emphasizes the importance of community partnerships, shared accountability, equity-oriented measurement, and sustained cross-sector coordination in supporting more integrated and responsive population health action (10, 15, 16).

### 3.1 Chronic disease prevention

Local assessments indicate that premature mortality within Shelby County remains above state and national averages, driven largely by hypertension, diabetes, and kidney disease (19–21, 23). Community health needs assessments and institutional reports describe a range of existing activities, including blood pressure control initiatives, diabetes education programs, nutrition support services, and efforts to increase opportunities for physical activity through community partnerships (24, 25, 29, 35). Within the proposed framework, these activities are organized under a shared population health priority focused on reducing preventable chronic disease morbidity and mortality through coordinated prevention, primary care integration, and community-centered intervention strategies.

The analysis also identified fragmentation across clinical, public health, and community-based efforts. Although multiple organizations address chronic disease prevention, activities frequently operate in parallel with limited integration of shared metrics, referral pathways, or cross-sector accountability structures. This limits the ability of health systems and community partners to address upstream determinants such as food insecurity, housing instability, transportation barriers, and neighborhood conditions that contribute to chronic disease inequities.

### 3.2 Policy and practice implications

Chronic disease prevention is more likely to achieve sustained population-level impact when prevention strategies are integrated across healthcare systems, public health agencies, and community-based organizations. Community partnerships can strengthen implementation by improving access to nutrition support, physical activity resources, and social services that address structural determinants of health. Embedding shared measurement frameworks, coordinated referral systems, and equity-focused indicators within primary care and community settings may help jurisdictions move from fragmented disease-specific programs toward coordinated, community-centered population health strategies (13, 40, 42).

### 3.3 Maternal and child health equity

Across local reports, infant mortality and preterm birth rates remain elevated, with substantial racial inequities; Black infants die at more than twice the rate of White infants within Shelby County (19–21, 23). Community health assessments and institutional reports identify barriers including limited access to comprehensive prenatal care, maternal stress, housing instability, transportation barriers, and inconsistent access to postpartum support services as important contributors to adverse maternal and infant outcomes (24, 25, 36, 37). Within the proposed framework, these findings are organized under a shared population health priority focused on maternal and child health equity, emphasizing the integration of clinical care, public health programming, and community-based social support systems.

The analysis identified persistent gaps between healthcare delivery and the broader social and structural conditions that influence maternal and infant health outcomes. Although many organizations provide maternal and child health services, coordination across healthcare systems, social services, housing programs, and community organizations remains inconsistent. Limited integration of shared metrics, referral pathways, and longitudinal support structures may reduce the effectiveness of existing interventions, particularly in communities experiencing concentrated socioeconomic disadvantage.

### 3.4 Policy and practice implications

Improving maternal and child health outcomes requires policy and practice approaches that extend beyond clinical services to address structural determinants of health, including housing stability, transportation access, paid leave, food security, and sustained social support during pregnancy and the postpartum period. Community partnerships and trusted local organizations may strengthen engagement and continuity of care by connecting families with culturally responsive services and longitudinal support systems. Coordinating maternal health initiatives with home visiting programs, behavioral health services, and community-based organizations can help jurisdictions move from fragmented interventions toward more integrated, prevention-oriented, and equity-centered population health strategies (3, 43).

### 3.5 HIV and sexual health

Local epidemiologic data indicate that Shelby County accounts for a disproportionate share of new HIV diagnoses within its state, with the highest rates occurring among Black men who have sex with men and Black heterosexual women (19–21, 23). Community health assessments and institutional reports identify ongoing needs related to expanded HIV testing, increased access to pre-exposure prophylaxis, linkage to longitudinal care, and stigma-free education delivered through trusted community organizations (26, 27, 44, 45). Within the proposed framework, HIV and sexual health are organized under a shared population health priority that integrates biomedical prevention, routine primary care, public health surveillance, and community-based outreach strategies.

The analysis identified persistent gaps between available biomedical advances and equitable access to prevention and care across affected communities. Although effective prevention and treatment tools exist, fragmentation across healthcare systems, public health programs, and community-based organizations may limit continuity of care, reduce uptake of preventive services, and contribute to disparities in viral suppression and long-term outcomes. Stigma, mistrust, transportation barriers, and inconsistent access to culturally responsive services further complicate engagement in prevention and treatment programs.

### 3.6 Policy and practice implications

Effective HIV prevention and care require coordinated strategies that integrate biomedical interventions with community-centered approaches addressing social and structural determinants of health. Partnerships between healthcare systems, public health agencies, and trusted community organizations are essential to expand access, reduce stigma, strengthen continuity of care, and improve engagement among disproportionately affected populations. Policy and practice strategies may include sustained funding for community partnerships, integration of sexual health services within primary care settings, expansion of culturally responsive outreach programs, and development of shared data systems that support routine monitoring of HIV testing, pre-exposure prophylaxis uptake, linkage to care, and viral suppression across populations and neighborhoods (42, 46).

### 3.7 Behavioral health and substance use

Local reports document increasing opioid and stimulant use, including substantial rises in overdose mortality over recent years, driven largely by synthetic opioids (20, 21, 23). Community health assessments identify persistent gaps in crisis response infrastructure, harm reduction services, treatment access, and integration of behavioral health within primary care settings (28, 29, 35, 36). Within the proposed framework, behavioral health and substance use are organized under a shared population health priority emphasizing trauma-informed care, harm reduction, coordinated crisis response, and stronger linkages between community-based services and clinical systems.

The analysis identified fragmentation across behavioral health, emergency response, public health, and primary care systems as a major barrier to continuity of care and sustained engagement in treatment. Individuals with substance use disorders often encounter disconnected services, inconsistent follow-up, limited treatment availability, and barriers related to transportation, housing instability, stigma, and insurance coverage. These system-level gaps may contribute to recurrent emergency department utilization, preventable overdose deaths, and widening inequities in behavioral health outcomes across communities.

### 3.8 Policy and practice implications

Reducing substance use–related morbidity and mortality requires coordinated population health strategies that integrate behavioral health services within primary care and strengthen connections with community-based outreach and support programs. Policy and practice approaches may include expansion of crisis response capacity, sustained investment in harm reduction programs, integration of behavioral health screening and treatment into routine clinical care, and reimbursement models that support collaborative and longitudinal care delivery. Strengthening partnerships among healthcare systems, public health agencies, emergency services, and community organizations may improve continuity of care, reduce fragmentation, and support more equitable access to prevention, treatment, and recovery services (42, 46).

### 3.9 Violence prevention and community safety

Local data sources identify injury and violence as major contributors to preventable mortality and community trauma. Homicide and assault-related injuries are disproportionately concentrated in neighborhoods experiencing persistent socioeconomic disadvantage, housing instability, and limited access to community resources (19–21, 23). Community health assessments and institutional reports describe a range of existing activities, including hospital-based violence intervention programs, youth development initiatives, trauma-informed services, and neighborhood-level safety partnerships involving community organizations and public agencies (28, 37, 38). Within the proposed framework, these activities are organized under a shared population health priority focused on violence prevention and community safety, linking healthcare systems, public health agencies, social services, schools, and community-based organizations.

The analysis identified substantial fragmentation across sectors involved in violence prevention, with programs often operating independently despite addressing interconnected social and structural drivers of violence. Existing efforts frequently lack shared governance structures, coordinated measurement strategies, and sustained mechanisms for community participation in decision-making. These gaps may limit the ability of local systems to address upstream determinants of violence and sustain long-term reductions in injury and trauma.

### 3.10 Policy and practice implications

Effective violence prevention requires coordinated, cross-sector strategies that integrate healthcare, public health, education, social services, and community organizations to address both immediate risks and the structural conditions associated with violence. Policy approaches may include development of formal cross-sector governance structures, shared indicators for violence-related outcomes, sustained investment in community-led prevention initiatives, and integration of trauma-informed approaches across health and social systems. Strengthening partnerships with affected communities and aligning prevention efforts across sectors may improve sustainability, accountability, and population-level impact (15, 41).

## 4 Actionable recommendations

Although the preceding domains reflect distinct epidemiologic patterns and operational challenges, several implementation needs emerged consistently across sectors. These included fragmented governance structures, limited interoperability between clinical and community-based systems, insufficient shared accountability mechanisms, and variability in community engagement processes. The following recommendations outline cross-cutting policy and practice strategies that may help jurisdictions strengthen coordination, improve implementation capacity, and support more durable and equity-oriented population health action.

### 4.1 Strengthening cross-sector governance

Jurisdictions seeking to improve population health outcomes should establish formal governance structures that align public health agencies, healthcare systems, community-based organizations, and local government partners around shared priorities. Many existing initiatives operate independently, resulting in fragmented implementation, duplication of services, and inconsistent accountability (5–7, 13, 14).

Cross-sector governance bodies may include regional population health collaboratives, interagency implementation councils, or shared steering committees responsible for coordinating strategic priorities and monitoring progress. Public health agencies are well positioned to serve as neutral conveners because of their statutory responsibility for population-level health assessment and prevention (8, 11, 13, 17). Governance structures should include community representation and clearly defined accountability mechanisms to sustain alignment across sectors despite changes in funding or leadership.

Collaborative governance models developed through accountable communities for health and collective impact initiatives suggest that shared decision-making structures may strengthen coordination between healthcare systems, public health agencies, and community organizations while improving accountability for population-level outcomes (16, 47).

### 4.2 Building shared data and measurement infrastructure

Effective population health strategy requires integrated approaches to data collection, sharing, and measurement. Current systems frequently separate clinical, public health, and social service data, limiting the ability to identify disparities, monitor implementation, and coordinate interventions across sectors (6, 13, 40).

Jurisdictions should prioritize interoperable data systems capable of linking clinical outcomes, public health surveillance indicators, and community-level measures related to housing, transportation, food access, and social vulnerability. Shared measurement frameworks should include indicators that can be routinely disaggregated by race, ethnicity, income, and geography to support equity-oriented decision-making (3, 13, 22, 40).

Investment in shared dashboards, data governance agreements, and regional analytic capacity may strengthen accountability and improve coordination across institutions participating in population health initiatives. Recent national efforts to modernize public health data systems following the COVID-19 pandemic have further highlighted the importance of integrated surveillance and interoperable infrastructure for population health planning (9).

### 4.3 Sustaining community partnerships and trust

Community partnerships should be treated as foundational infrastructure rather than supplemental engagement activities. Community-based organizations and affected communities possess contextual knowledge that is essential for identifying implementation barriers, improving program acceptability, and sustaining trust in public health initiatives (8, 14).

Long-term partnership strategies should include shared decision-making processes, sustained funding mechanisms for community organizations, and inclusion of community representatives within governance structures. Particular attention should be given to historically marginalized populations that may have experienced distrust related to prior public health or healthcare interventions.

The COVID-19 pandemic reinforced the importance of trust as a determinant of public health effectiveness, particularly in vaccination efforts, risk communication, and uptake of preventive services. Trust-building requires continuity, transparency, and visible responsiveness to community priorities rather than episodic engagement tied only to grant cycles or assessment requirements (10, 31, 32). Community health workers, faith-based organizations, and neighborhood coalitions may play critical roles in strengthening trust and improving implementation within underserved communities (42, 46).

### 4.4 Aligning financing and institutional incentives

Many health systems continue to operate within financing structures that prioritize clinical service delivery over prevention and cross-sector collaboration. This misalignment can limit investment in community-based prevention strategies and upstream interventions addressing social determinants of health (5, 7).

Population health initiatives may be strengthened through financing mechanisms that support shared accountability across sectors, including value-based payment arrangements, braided funding models, and joint investment strategies between healthcare systems and public health agencies. Alignment between institutional performance metrics and community-level outcomes may also encourage greater integration of prevention activities within healthcare delivery systems (13, 40, 42).

Nonprofit hospital community benefit requirements and emerging accountable care models may provide opportunities to redirect investment toward community-based prevention and population health infrastructure (48, 49). Operational alignment should extend to staffing models, strategic planning, and evaluation processes so that population health goals are incorporated into routine organizational decision-making rather than treated as parallel initiatives.

### 4.5 Supporting long-term public health infrastructure

The COVID-19 pandemic highlighted longstanding weaknesses in public health infrastructure, including workforce shortages, fragmented data systems, and unstable funding. Sustainable population health improvement requires continued investment in foundational public health capacity beyond periods of acute crisis.

Long-term infrastructure priorities include workforce development, epidemiologic and analytic capacity, interoperable information systems, and sustained support for prevention-oriented partnerships across sectors. Jurisdictions should also strengthen mechanisms for translating community health assessment findings into operational planning, budgeting, and implementation processes (8, 11, 13).

Recent national reports have emphasized that durable public health infrastructure depends on sustained investment rather than episodic emergency funding tied to public health crises (9, 50). Frameworks such as the one presented here may help jurisdictions move from episodic assessment activities toward more durable and coordinated population health strategies capable of adapting to changing community needs and policy environments.

## 5 Discussion

This analysis demonstrates how publicly available community health data can be translated into a population health framework that clarifies priorities, identifies system gaps, and supports coordinated policy decision-making. Using Shelby County, Tennessee, as an illustrative case, the findings suggest that many barriers to improving population health arise not from a lack of programs, but from the absence of shared strategy, governance, and measurement across sectors (15, 16, 19–21, 23). Similar challenges have been documented in other jurisdictions, where fragmented service delivery and limited data integration constrain the impact of community health assessments and improvement efforts (7, 8, 14). This framework attempts to address the persistent implementation gap between community health assessment and coordinated population health action.

Several features of the framework support its relevance beyond the local context. Grounding priority-setting in local epidemiologic and contextual data may help establish legitimacy across institutions that often operate in parallel and with differing mandates (12, 13, 19, 40). Positioning public health agencies as conveners and community organizations as co-producers of health is consistent with literature on multisector partnerships, integrated care, and collective impact approaches (15, 16, 27, 28, 33, 41). The emphasis on shared measurement and accountability reflects growing recognition that coordinated indicators and evaluation cycles are necessary to move from descriptive assessment toward sustained, equity-oriented action (6, 7, 9, 14, 15, 42).

This work has several limitations. It describes an analytic framework rather than an implementation study, and the proposed approach has not been tested in practice. Future application would require collaboration among public health agencies, healthcare systems, and community partners to determine feasibility, resource requirements, and governance structures (5, 46). Implementation of this framework would likely require dedicated personnel, sustained stakeholder engagement, and cross-sector organizational commitment. While initial data review and framework development could potentially be led by a small public health team, successful implementation would depend on active participation from healthcare systems, public health agencies, community-based organizations, and community representatives. Depending on local capacity and data availability, jurisdictions may require several months to complete assessment, priority-setting, governance development, and indicator selection before implementation activities begin. The framework does not require specialized software and can be operationalized using publicly available datasets, standard spreadsheet applications, and commonly used data visualization platforms. However, jurisdictions with access to interoperable data systems, analytic expertise, and established cross-sector partnerships may be better positioned to support ongoing monitoring, evaluation, and continuous improvement.

An additional implementation challenge is that healthcare system catchment areas, public health jurisdictions, and community-defined boundaries do not always align. Many healthcare organizations serve populations that span multiple counties or regions, whereas publicly available health indicators are often reported at county, state, or other administrative levels. Data availability, reporting frequency, and geographic granularity may therefore limit the ability to characterize communities consistently or monitor outcomes across all populations served. Defining the relevant population for assessment and action may require adaptation of the framework to local circumstances and available data sources.

Potential improvements across domains, including chronic disease, maternal and child health, behavioral health, and violence prevention, remain hypothetical until jurisdictions apply the framework using longitudinal indicators disaggregated by race, income, and geography to assess equity effects (3, 43). In addition, variation in political context, institutional capacity, and cross-sector readiness may require adaptation for use in other settings (8, 22). Implementation may also be constrained by fragmented governance structures, limited data-sharing capacity, and variability in institutional readiness across jurisdictions.

Despite these limitations, the framework contributes to ongoing policy discussions on how jurisdictions can translate assessment findings into coordinated, equity-centered population health strategies. Figure 1 provides a practical scaffold illustrating how assessment of population health data, cross-sector stakeholder engagement, definition of shared priorities, alignment of governance and system capacity, and implementation, monitoring, and evaluation can be organized into an iterative process responsive to changing population needs (4, 6, 42). Centering equity, community voice, and accountability underscores the importance of evaluating not only average outcomes, but also their distribution across populations and neighborhoods (1, 3, 4, 10, 32, 42).

## 6 Conclusion

Population health strategies must remain durable across changing political and organizational environments. Short-term initiatives often shift with leadership transitions, funding cycles, or policy priorities. A clearly articulated population health framework can provide continuity of purpose, helping jurisdictions sustain long-term commitments to prevention, equity, and community wellbeing even as specific programs evolve.

The framework presented here offers a structured approach for translating local data, system capacity, and community priorities into coordinated population health action. Although further implementation and evaluation are needed, the framework provides a practical foundation for jurisdictions seeking to strengthen cross-sector alignment, embed equity into planning and evaluation, and move from episodic assessment toward sustained and measurable improvements in population health.
